# Contrast-Enhanced Mammography vs. Breast MRI for Assessing Neoadjuvant Chemotherapy Response: A Prospective Clinical Comparison Study

**DOI:** 10.3390/diagnostics16040640

**Published:** 2026-02-23

**Authors:** Omer Acar, Çağdaş Rıza Açar, İhsan Sebnem Orguc, Ferhat Ekinci, Mustafa Sahbazlar, Atike Pınar Erdoğan

**Affiliations:** 1 Department of Medical Oncology, Mardin Training and Research Hospital, Mardin 47200, Turkey; 2 Department of Radiology, Manisa Celal Bayar University, Manisa 45030, Turkey; dr.cagdasriza@gmail.com (Ç.R.A.); sebnemorguc@hotmail.com (İ.S.O.); 3 Department of Medical Oncology, Manisa Celal Bayar University, Manisa 45030, Turkey; drferhatekinci@hotmail.com (F.E.); dr_pinarcan@yahoo.com (A.P.E.)

**Keywords:** CEM, MRI, NAC, breast cancer

## Abstract

**Objective**: To compare contrast-enhanced mammography (CEM) with breast magnetic resonance imaging (MRI) in evaluating residual tumor size and pathological complete response after neoadjuvant chemotherapy (NAC) in breast cancer patients. **Methods**: This prospective study included patients with histopathologically confirmed breast cancer who were scheduled to receive NAC followed by surgery. All patients underwent both CEM and breast MRI before initiation of NAC and within seven days after completion of treatment. Surgery was performed at a median of 14 days after post-treatment imaging. Residual tumor size measurements obtained by both imaging modalities were compared with histopathological findings, which served as the reference standard. Pathological complete response was defined as the absence of residual invasive carcinoma in the surgical specimen. **Results**: A total of 74 female patients were included. CEM estimated residual tumor size within ±1 cm of histopathology in 84.7% of cases, whereas MRI achieved this accuracy in 76.4%. Agreement with histopathology was higher for CEM than for MRI. In predicting pathological complete response, CEM demonstrated higher sensitivity (91.3%) and negative predictive value compared with MRI; however, this difference did not reach statistical significance (*p* = 0.24). MRI showed slightly higher specificity. Pathological complete response was observed in 31.1% of patients. **Conclusion**: Contrast-enhanced mammography demonstrated performance comparable to breast MRI in assessing response to neoadjuvant chemotherapy, with numerically higher sensitivity for predicting pathological complete response. CEM may represent a practical and accessible alternative to MRI, particularly in settings where MRI is unavailable or contraindicated. These findings support the clinical use of CEM as a reliable alternative imaging modality for response assessment.

## 1. Introduction

Neoadjuvant chemotherapy (NAC) has become a standard treatment strategy for patients with stage II–III breast cancer. In addition to tumor downstaging, NAC enables in vivo assessment of treatment sensitivity and may increase the likelihood of breast-conserving surgery while reducing the need for axillary dissection [1]. Achieving a pathological complete response (pCR) after NAC is strongly associated with improved long-term survival outcomes, particularly in biologically aggressive subtypes such as triple-negative and HER2-positive breast cancer [2]. Imaging techniques are used before and after NAC to assess chemosensitivity and assist surgical planning. Post-NAC clinical examination, mammography, ultrasound, and breast magnetic resonance imaging (MRI) help predict pathological complete response. Breast MRI is widely considered the most reliable imaging modality for estimating residual tumor burden and predicting pathological response after NAC [3,4,5]. However, it is contraindicated for patients with metallic implants and pacemakers. Additionally, some patients experience anxiety from claustrophobia or noise during MRI scans. It may also be less preferred due to its lengthy scanning time.

Contrast-enhanced mammography (CEM) is a relatively affordable and convenient new imaging method that involves administering an enhanced iodinated contrast agent using the dual-energy technique to evaluate tumor neovascularity. It offers higher diagnostic sensitivity than digital mammography and provides more accurate estimates of lesion sizes [6]. CEM provides both morphological and functional information through contrast enhancement, offering diagnostic capabilities comparable to MRI in several clinical scenarios [7]. The aim of this study was to compare the diagnostic performance of CEM and MRI in assessing residual tumor size and predicting pCR after NAC, using histopathology as the reference standard.

## 2. Material and Method

### 2.1. Study Design and Setting

This prospective clinical study was conducted at Manisa Celal Bayar University Hafsa Sultan Hospital between 2022 and 2024. All participants provided written informed consent before enrollment.

### 2.2. Patient Selection and Eligibility Criteria

Patients older than 18 years with histopathologically confirmed breast cancer who indicated NAC and no contraindications for both CEM and MRI were included.

Exclusion criteria were history of allergy to iodinated or gadolinium-based contrast agents, renal failure, pregnancy or suspected pregnancy, claustrophobia, body weight incompatible with the MRI scanner, and presence of metallic implants or pacemakers.

### 2.3. Neoadjuvant Chemotherapy Protocols

HER2-negative patients received four cycles of dose-dense anthracycline (doxorubicin 60 mg/m^2^ plus cyclophosphamide 600 mg/m^2^ every two weeks), followed by paclitaxel 80 mg/m^2^ weekly for 12 weeks or paclitaxel 175 mg/m^2^ every two weeks, depending on patient preference, during the COVID-19 pandemic. Triple-negative patients received paclitaxel 80 mg/m^2^ plus carboplatin (AUC 2) following anthracycline therapy. HER2-positive patients were treated with either THP (docetaxel + trastuzumab + pertuzumab) or TCHP (docetaxel + carboplatin + trastuzumab + pertuzumab) regimens.

### 2.4. Imaging Protocols

All patients underwent CEM and MRI examinations both before NAC and within 7 days of NAC completion.

#### 2.4.1. Contrast-Enhanced Mammography (CEM)

CEM was performed using a dual-energy digital mammography system (Pristina, GE Healthcare, Reu De La Miniere, Buc, France). An iodinated contrast agent was injected intravenously at 1.5 mL/kg (maximum 100 mL), and low- and high-energy CC and MLO images were obtained 1.5 min after injection. Scanning time was approximately 5 min.

#### 2.4.2. Breast Magnetic Resonance Imaging (MRI)

MRI examinations were performed using a 1.5-Tesla system (Signa HDx; GE Healthcare, Milwaukee, WI, USA). T1- and T2-weighted, DWI, and dynamic contrast-enhanced sequences were acquired after intravenous gadolinium-based contrast administration (0.1 mmol/kg). Subtraction imaging was used to evaluate lesion enhancement.

### 2.5. Imaging Evaluation and Response Assessment

All images were interpreted by two breast radiologists (32 and 5 years of experience).

The largest diameter of the enhancing lesion was recorded for each modality.

In multifocal disease, the largest lesion was used for analysis.

Therapeutic response was classified according to RECIST version 1.1: complete response (CR), partial response (PR), stable disease (SD), progressive disease (PD) [8]. Pathological complete response (pCR) was defined as the absence of residual invasive carcinoma in the surgical specimen; cases with residual DCIS were considered pCR. Surgery was performed within a median of 14 days (IQR: 10–18 days) after completion of post-NAC imaging.

### 2.6. Outcomes

The primary outcome was the accuracy of residual tumor size measurement compared with pathology. The secondary outcomes were diagnostic performance of CEM and MRI in predicting pCR.

### 2.7. Statistical Analysis

SPSS 15.0 for Windows and MedCalc 15.2 package were used for statistical analysis. Descriptive statistics were presented as mean, standard deviation, minimum, maximum, and median for numeric variables, as well as count and rate for categorical variables. The relationships between measurements were examined using Bland–Altman plots, Lin’s concordance, and bivariate Pearson correlation analysis. Accuracy was defined as agreement between imaging-estimated and pathological tumor size within predefined thresholds (±1.0 cm and ±0.5 cm). A *p*-value < 0.05 was considered statistically significant.

### 2.8. Ethical Approval

This study was approved by the Health Sciences Ethics Committee of the Faculty of Medicine, Manisa Celal Bayar University (Decision No: 227, Date: 2 December 2021).

## 3. Results

### 3.1. Patient Characteristics

A total of 74 female patients with a median age of 48 years (range 29–75) were included. Most tumors were invasive ductal carcinoma (93.2%) and clinical stage II at presentation (68.9%). Molecular subtypes were Luminal B in 47.3%, triple-negative in 20.3%, Luminal A in 25.7%, and HER2-positive/ER-negative in 6.8% of patients. Mastectomy was performed in 78.4% of patients (Table 1).

### 3.2. Accuracy of Tumor Size Measurement

The mean baseline tumor size before NAC was 37.2 ± 22.3 mm on CEM and 38.4 ± 21.7 mm on MRI. The mean residual invasive tumor size at final pathological examination was 11.7 mm. Post-NAC mean tumor size was 10.7 ± 15.2 mm with CEM and 13.3 ± 16.4 mm with MRI. Residual tumor size was measured within ±1 cm accuracy in 84.7% of cases with CEM and 76.4% with MRI. Overestimation rates were 6.9% for CEM and 15.3% for MRI, while the underestimation rate was equal in both modalities (8.3%) (Table 2 and Table 3).

In addition to the predefined ±1.0 cm threshold, we performed a secondary analysis using a stricter ±0.5 cm criterion. As expected, narrowing the threshold reduced the accuracy of both modalities. Using the ±0.5 cm criterion, CEM correctly estimated residual tumor size in 68.1% of cases (49/72), whereas MRI achieved 58.3% accuracy (42/72). Although CEM maintained numerically higher accuracy, the paired comparison did not reach statistical significance (exact McNemar *p* = 0.118). Detailed results are presented in Supplementary Table S1.

**Figure 1:**
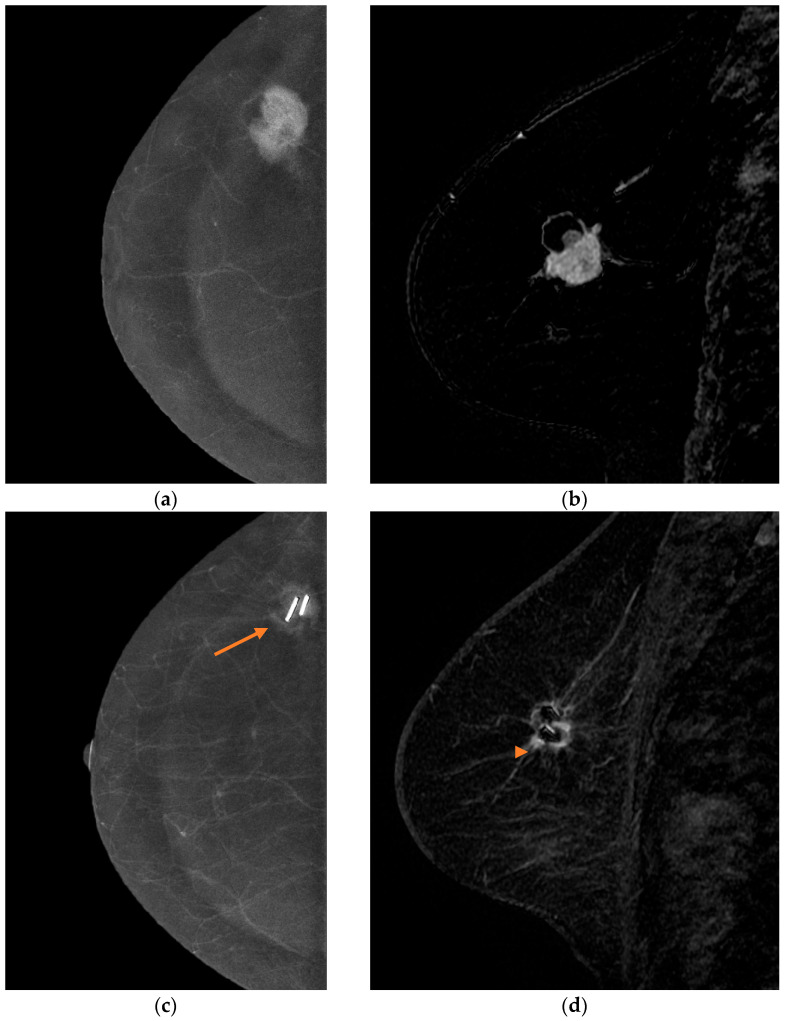
Contrast-enhanced mammography and MRI findings before and after treatment in a 56-year-old woman with invasive ductal carcinoma. Pre-treatment recombined CEM image demonstrates an irregular, heterogeneously enhancing mass in the right breast (**a**). Corresponding contrast-enhanced T1-weighted MRI confirms avid enhancement of the same lesion (**b**). Post-treatment recombined CEM image demonstrates marked tumor regression with residual moderate enhancement at the original tumor site, marked by stereotactic localization markers (arrow) (**c**). Corresponding post-treatment MRI demonstrates residual enhancement and decreased lesion size, with susceptibility artifact related to the markers (arrowhead) (**d**). Residual tumor size measured 18 mm on CEM and MRI, while histopathology demonstrated an 8 mm residual invasive tumor.

A representative case demonstrating tumor size estimation and residual disease assessment using both modalities is shown in Figure 1, supporting the observed quantitative agreement among CEM, MRI, and pathology.

### 3.3. Agreement Between Imaging Methods and Pathology

CEM showed stronger concordance with pathology compared to MRI.

Post-NAC Lin’s concordance coefficients: CEM vs. pathology: 0.77, MRI vs. pathology: 0.65.

Bland–Altman analysis demonstrated a statistically significant difference between CEM and MRI in post-treatment tumor size measurements (*p* = 0.0085). This finding suggests a systematic measurement difference between the two modalities, with CEM showing closer agreement with pathological tumor size (Table 4 and Table 5).

### 3.4. Prediction of Pathological Complete Response (pCR)

Pathological complete response was achieved in 23 patients (31.1%).

CEM demonstrated numerically higher sensitivity in predicting pCR compared with MRI:

- Sensitivity: 91.3% vs. 73.9%;
- Specificity: 70.6% vs. 74.5%;
- Negative predictive value: 94.7% vs. 86.4%;
- Positive predictive value: 58.3% vs. 56.7% (Table 6).

Additional subgroup analyses, stratified by menopausal status, tumor stage, molecular subtype, histology, and BRCA mutation status, are presented in Supplementary Table S2. Across subgroups, CEM showed numerically higher sensitivity in several categories, while specificity was generally comparable between CEM and MRI. No statistically significant differences were observed in paired comparisons.

## 4. Discussion

In locally advanced breast cancer, imaging is essential for treatment planning before and after NAC. Even when a complete radiological response is observed at the end of NAC, surgical treatment remains recommended per current clinical guidelines. MRI is widely regarded as the most reliable imaging modality for clinical diagnosis, staging, follow-up, and monitoring response to NAC in breast cancer [3,5,9]. New imaging techniques are essential because MRI is contraindicated in patients with metallic implants, has a long procedure time, and can cause claustrophobia and anxiety in some patients. Recently, CEM—a new imaging technique that combines morphologic and tumor neoangiogenesis information—has been reported to perform at least as well as MRI [10]. CEM’s greater accessibility, lower cost, and higher tolerability compared to MRI have made it a suitable alternative [11,12]. Additionally, CEM has demonstrated superiority over MRI by using both microcalcifications (on the low-energy image) and contrast-enhancing structures (on the recombined image) in a single exam [13,14].

Most imaging techniques tend to overestimate or underestimate tumor size. Several factors, including pre-treatment tumor size, parenchymal distortion and asymmetry, calcification, edema, necrosis, histological subtype, and NAC selection, influence tumor shrinkage. The decrease in tumor cells after chemotherapy may not be directly proportional to the reduction in tumor size. Even if tumor cells are destroyed, fibrous stroma may remain in the tumor bed. These issues make it difficult to predict the pathological response [15,16].

In our study, CEM accurately measured tumor size, closely matching the histopathological size. In nearly 85% of cases, the difference between CEM and histopathological tumor size was within 1 cm. This rate exceeds that of MRI, which has been regarded as the gold standard imaging method so far (MRI 76.4%). In a Brazilian study with a design similar to ours, the proportion of patients with a difference between CEM and histopathological tumor size of less than 1 cm was 70% [17]. More recent studies have also compared CEM and MRI in evaluating residual disease after neoadjuvant chemotherapy and have reported comparable diagnostic performance between the two modalities [15,18,19,20,21,22,23]. In our study, there was very strong agreement between the pathological residual tumor and CEM. The Lin’s correlation coefficient and Pearson correlation coefficient were higher for CEM than for MRI (Lin’s correlation coefficients 0.77 vs. 0.65, respectively). Studies conducted in the USA, Brazil, and Italy have reported that the Lin’s correlation coefficients for CEM are similar, close to 0.8 [17,24,25]. In our study, the correlation coefficient between CEM and MRI before NAC was 0.92, but it dropped to 0.85 after NAC. Similarly, in the study by Lotti et al., Lin’s correlation concordance between CEM and MRI was highest before NAC but declined after NAC [25]. The mean difference in tumor sizes between CEM and MRI before treatment was 0.6 cm, increasing to 2.6 cm after NAC. According to Bland–Altman plots, this difference is statistically significant. These plots suggest closer agreement between CEM and pathological measurements, particularly following NAC. The median imaging-to-surgery interval was approximately two weeks; therefore, although additional tumor regression cannot be entirely excluded, both imaging modalities were performed within the same timeframe and would have been affected similarly by this interval. When a stricter ±0.5 cm threshold was applied, accuracy decreased for both modalities, as expected. CEM remained numerically higher than MRI at both thresholds; however, the differences were not statistically significant.

CEM demonstrated higher sensitivity than MRI in identifying patients with histopathological complete response; however, this difference did not reach statistical significance (χ^2^ *p* = 0.24). In our study, sensitivity, PPV, and NPV were numerically higher for CEM, whereas MRI showed a slightly higher specificity (74.5% vs. 70.6%). Importantly, the observed difference in specificity between CEM and MRI was modest and did not translate into a statistically significant overall difference in diagnostic performance. Although several previous studies have reported comparable or even superior specificity for CEM, our findings demonstrated a modestly lower specificity than MRI. This discrepancy may be related to differences in study populations, tumor biology distribution, and sample size. In particular, the predominance of invasive ductal carcinoma in our cohort and the relatively high pCR prevalence may have influenced specificity estimates. Furthermore, small residual microscopic tumor foci with limited vascular perfusion may not produce significant enhancement on CEM, potentially contributing to misclassification and reduced specificity [21,26,27].

Given the known variability in pCR rates among molecular subtypes, we additionally performed a subgroup analysis focusing on Luminal A tumors, which represented 25.7% of our cohort. As expected, none of the Luminal A patients achieved pathological complete response. Therefore, sensitivity could not be meaningfully calculated in this subgroup. However, both CEM and MRI demonstrated identical specificity rates (84.2%) among Luminal A patients, indicating comparable performance in correctly identifying residual disease in this biologically less chemosensitive subgroup. This finding suggests that the overall pCR rate observed in our cohort was not driven by Luminal A tumors and therefore did not bias the comparative performance analysis between CEM and MRI.

Previous studies have reported heterogeneous findings. Lotti et al. observed higher sensitivity, specificity, NPV, and PPV for CEM compared to MRI [25]. Patel et al. reported nearly similar diagnostic performance between CEM and MRI [24], whereas Barra et al. found higher sensitivity and NPV for MRI but higher specificity and PPV for CEM [17]. Similarly, Elsaid et al. reported a sensitivity of 100% and a specificity of 83% for CEM in predicting pathological complete response in a smaller cohort of 21 patients [28]. In our study, CEM demonstrated high sensitivity in identifying patients with pCR, although this difference was not statistically significant. However, many patients showed a complete response on CEM imaging but did not have a histopathological complete response (PPV: 58.3%). This may be explained by the presence of small residual tumor foci within the tumor bed that do not demonstrate sufficient contrast enhancement, potentially leading to false-positive interpretations of complete response on CEM. Previous studies and our own research have shown that CEM can be used as an alternative to or alongside MRI. The image acquisition time for CEM ranges from 7 to 10 min, which is significantly shorter than the over 30 min typically required for MRI. In addition to increasing patient comfort, this also allows radiologists to utilize their time more efficiently. However, CEM does have some drawbacks compared to MRI. MRI has the benefit of imaging the entire chest wall and axilla region. It also does not use ionizing radiation and does not require compression. Although iodinated contrast agents are generally considered more hazardous than gadolinium contrast agents, the health effects of gadolinium accumulation are still controversial. Although breast MRI is recommended as the imaging method for dense breasts, CEM has also performed well in this patient group. Allergic reactions, extravasation, and contrast nephropathy have rarely occurred in patients given intravenous contrast for CEM. However, most side effects were mild and resolved on their own.

The relatively high mastectomy rate observed in our cohort may be attributed to initial tumor stage, multifocal disease, tumor bed extent, patient preference, and institutional practice patterns during the study period. It should be noted that the primary aim of this study was to evaluate imaging accuracy rather than to assess changes in surgical management strategies.

Our study has some limitations. Although we performed a subgroup analysis for Luminal A tumors, the limited sample size and absence of pCR events in this subgroup restricted more detailed statistical comparisons. Larger studies stratified by molecular subtypes are needed to further clarify potential differences in imaging performance. Since pCR rates differ substantially among subtypes, particularly in Luminal A tumors where response rates are typically lower, imaging performance may also vary across biological subgroups [29]. Larger studies are needed to evaluate diagnostic accuracy stratified by molecular subtype. We were unable to assess the effect of CEM for different histological subtypes, because the study population included only 2 lobular cancer patients. MRI is considered the most accurate imaging tool for diagnosing and staging invasive lobular cancer [30]. In a study conducted on a small number of patients with invasive lobular cancer, CEM showed performance similar to MRI [18]. Therefore, studies with a larger patient population are needed regarding applicability in invasive lobular cancer patients and high-risk patients such as those with BRCA mutations. Additionally, digital breast tomosynthesis was not incorporated into the CEM protocol in this study. Future studies evaluating the potential contribution of tomosynthesis to CEM may help determine whether combined imaging further improves response assessment accuracy.

## 5. Conclusions

In patients receiving neoadjuvant chemotherapy, contrast-enhanced mammography demonstrated diagnostic performance comparable to breast MRI in assessing residual tumor size. CEM showed numerically higher sensitivity for predicting pathological complete response; however, this difference did not reach statistical significance (χ^2^ *p* = 0.24). Overall, CEM may represent a practical and accessible alternative to MRI, particularly in settings where MRI is unavailable or contraindicated. Further larger-scale studies are warranted to confirm these findings.

## Figures and Tables

**Table 1:** Baseline clinicopathological characteristics of the study population.

Age Mean ± SD (Min–Max/Median)		48.5 ± 11.4 (29–75/48)
BRCA mutation *n* (%)	No	14 (18.9)
	Yes	9 (12.2)
	Unknown	51 (68.9)
Familial History *n* (%)	No	54 (73.0)
	Yes	20 (27.0)
Menopausal Status *n* (%)	Pre or Perimenopausal	41 (56.2)
	Postmenopausal	32 (43.8)
Molecular Characteristics *n* (%)	Luminal A	19 (25.7)
	Luminal B	35 (47.3)
	Her2 + Hormone Negative	5 (6.8)
	Triple Negative	15 (20.3)
Histological Subtype *n* (%)	Invasive Ductal	69 (93.2)
	Invasive Lobular	2 (2.7)
	Other	3 (4.1)
Location *n* (%)	Right Breast	37 (50.0)
	Left Breast	36 (48.6)
	Bilateral	1 (1.4)
Pre-Treatment Clinical Stage *n* (%)	Stage 1	5 (6.8)
	Stage 2	51 (68.9)
	Stage 3	18 (24.3)
Operation Type *n* (%)	Total Mastectomy	58 (78.4)
	Lumpectomy	16 (21.6)

BRCA: Breast cancer gene (tumor suppressor gene involved in DNA repair).

**Table 2:** Mean tumor size measurements before and after neoadjuvant chemotherapy.

	Mean ± SD (Min–Max/Median)
Pre-CEM size	37.2 ± 22.3 (8–110/30)
Pre-MRI size	38.4 ± 21.7 (12–105/31.5)
Post-CEM size	10.7 ± 15.2 (0–70/4)
Post-MRI size	13.3 ± 16.4 (0–65/10)
Tumor bed size	37.2 ± 24.5 (6–105/30)
Residual tumor	11.4 ± 15.8 (0–94/5)

CEM: Contrast-enhanced mammography; MRI: magnetic resonance imaging; Pre: before neoadjuvant treatment imaging; Post: after neoadjuvant treatment imaging.

**Table 3:** Accuracy of CEM and MRI in estimating residual tumor size within ±1 cm of pathology.

	*n* (%)
CEM vs. pathology	
CEM overestimate pathology (>1 cm)	5 (6.9)
CEM underestimate pathology (<1 cm)	6 (8.3)
CEM within 1 cm	61 (84.7)
MRI vs. pathology	
MRI overestimate pathology (>1 cm)	11 (15.3)
MRI underestimate pathology (<1 cm)	6 (8.3)
MRI within 1 cm	55 (76.4)
CEM vs. MRI	
CEM greater than MRI (>1 cm)	-
CEM less than MRI (<1 cm)	8 (10.8)
CEM within 1 cm vs. MRI	66 (89.2)

CEM: contrast-enhanced mammography; MRI: magnetic resonance imaging; equivalence criteria: imaging measurements within ±1 cm of pathological tumor size were considered accurate.

**Table 4:** Concordance and correlation analysis of tumor size measurements compared with pathology.

	Concordance Correlation Coefficient (95% CI)	Pearson Correlation
Pre-CEM size vs. pre-MRI size	0.917 (0.869 to 0.948)	0.919
Pre-CEM size vs. tumor bed size	0.728 (0.591 to 0.824)	0.731
Pre-MRI size vs. tumor bed size	0.705 (0.554 to 0.811)	0.708
Post-CEM size vs. post-MRI size	0.848 (0.771 to 0.901)	0.862
Post-CEM size vs. residual tumor	0.769 (0.655 to 0.849)	0.770
Post-MRI size vs. residual tumor	0.653 (0.499 to 0.767)	0.658

CEM: Contrast-enhanced mammography; MRI: magnetic resonance imaging; Pre: before neoadjuvant treatment imaging; Post: after neoadjuvant treatment imaging; CI: confidence interval.

**Table 5:** Bland–Altman analysis for agreement between imaging modalities and pathology.

	Mean = 0 Arithmetic Mean	95% CI	*p* (H_0_: Mean = 0)
Pre-CEM size vs. pre-MRI size	−0.6	−2.8 to 1.6	0.608
Pre-CEM size vs. tumor bed size	−0.2	−4.4 to 4.1	0.943
Pre-MRI size vs. tumor bed size	1.0	−3.6 to 5.6	0.669
Post-CEM size vs. post-MRI size	−2.64	−4.6 to −0.7	0.0085
Post-CEM size vs. residual tumor	−0.7	−3.2 to 1.8	0.572
Post-MRI size vs. residual tumor	1.9	−1.3 to 5	0.235

CEM: contrast-enhanced mammography; MRI: magnetic resonance imaging; Pre: before neoadjuvant treatment imaging; Post: after neoadjuvant treatment imaging; CI: confidence interval; bias values represent the mean difference between imaging and pathological measurements; *p*-value < 0.05 was considered statistically significant. Negative mean values indicate that imaging underestimated tumor size compared with pathology.

**Table 6:** Diagnostic performance of CEM and MRI in predicting pathological complete response.

		Histopathology pCR	Histopathology Non-pCR (pPR, pSD, pPD)	
CEM	pCR	21	15	PPV 58.3% (CI 47.3–68.6%) PPV 94.7% (CI 82.6–98.6%)
	Non-CR (PR, SD, PD)	2	36	
		Sensitivity 91.3% (CI 72–98.9%)	Specificity 70.6% (CI 56.2–82.5%)	
MRI	pCR	17	13	PPV 56.7% (CI 43.5–68.9%) PPV 86.4% (CI 75.8–92.8%)
	Non-CR (PR, SD, PD)	6	38	
		Sensitivity 73.9% (CI 51.6–89.8%)	Specificity 74.5% (CI 60.4–85.7%)	

CEM: contrast-enhanced mammography; MRI: magnetic resonance imaging; PR: partial response; SD: stable disease; PD: progressive disease; pCR: pathologic complete response; pPR: pathologic partial response; pSD: pathologic stable disease; pPD: pathologic progressive disease; PPV: positive predictive value; NPV: negative predictive value.

## Data Availability

The datasets generated and/or analyzed during the current study are not publicly available, due to patient confidentiality and institutional data protection policies, but are available from the corresponding author on reasonable request.

## Supplementary Materials

The following supporting information can be downloaded at: https://www.mdpi.com/article/10.3390/diagnostics16040640/s1, Table S1: Accuracy of CEM and MRI in estimating residual tumor size using ±1.0 cm and ±0.5 cm thresholds (reference standard: pathology), Table S2: Subgroup analysis of diagnostic performance of CEM and MRI in predicting pathological complete response (pCR).
