# Supplementary material for: Contrast-Enhanced Mammography vs. Breast MRI for Assessing Neoadjuvant Chemotherapy Response: A Prospective Clinical Comparison Study
Source: Diagnostics (Basel). 2026 Feb 23;16(4):640. doi: 10.3390/diagnostics16040640 (PMC12938973; doi:10.3390/diagnostics16040640)
Supplement: Supplementary file 1 [file diagnostics-16-00640-s001.zip › diagnostics-4127868-supplementary.pdf]

Supplementary Table S1. Accuracy of CEM and MRI in estimating residual tumor size using ±1.0 cm and ±0.5 cm thresholds (reference standard: pathology).

Threshold (vs pathology)	n	CEM Correct n (%)	MRI Correct n (%)	Δ Accuracy (CEM–MRI, %)	Exact McNemar p
±1.0 cm (≤10 mm)	72	61 (84.7%)	55 (76.4%)	+8.3	0.109
±0.5 cm (≤5 mm)	72	49 (68.1%)	42 (58.3%)	+9.8	0.118

Abbreviations: CEM, contrast-enhanced mammography; MRI, magnetic resonance imaging. Accuracy was defined as concordance between imaging-estimated residual tumor size and pathological measurement within the specified threshold. Paired comparisons were performed using the exact McNemar test. As the threshold was narrowed from ±1.0 cm to ±0.5 cm, accuracy decreased for both modalities; however, CEM remained numerically higher than MRI at both thresholds, without reaching statistical significance.

Supplementary Table S2. Subgroup analysis of diagnostic performance of CEM and MRI in predicting pathological complete response (pCR).

Subgroup	n	pCR (n)	CEM Sens (%)	CEM Spec (%)	MRI Sens (%)	MRI Spec (%)
**Overall**	74	23	91.3	70.6	73.9	74.5
**Premenopausal**	41	15	86.7	61.5	60.0	69.2
Postmenopausal	32	8	100	80.0	100	80.0
**Stage I–II**	56	18	94.4	65.8	72.2	73.7
Stage III	18	5	80.0	84.6	80.0	76.9
**Luminal A**	19	0	NA	84.2	NA	84.2
Luminal B	35	11	90.9	62.5	72.7	70.8
Triple-negative	15	7	100	62.5	85.7	62.5
HER2+/ER–	5	5	80.0	NA	60.0	NA
**Histology – IDC**	69	23	91.3	67.4	73.9	71.7
Histology – Other	5	0	NA	100	NA	100
**BRCA – Mutant**	15	5	100	60.0	60.0	80.0
BRCA – Wild-type	8	4	100	50.0	75.0	50.0
BRCA – Unknown	51	14	85.7	77.0	78.6	76.2

Abbreviations: CEM, contrast-enhanced mammography; MRI, magnetic resonance imaging; pCR, pathological complete response; IDC, invasive ductal carcinoma. Sensitivity and specificity were calculated using pathology as the reference standard. NA indicates that sensitivity or specificity could not be calculated, because the respective subgroup lacked pCR or non-pCR cases. Subgroup analyses were descriptive and not powered for statistical comparison.
